# Influenza in Tropical Regions

**DOI:** 10.1371/journal.pmed.0030089

**Published:** 2006-03-07

**Authors:** Cécile Viboud, Wladimir J Alonso, Lone Simonsen

## Abstract

Viboud and colleagues discuss the implications of a new study by Peiris et al. that assesses the burden of influenza in Hong Kong.

The global burden of influenza on morbidity and mortality is considerable, with an estimated 1 million annual deaths worldwide [ 1]. In temperate regions, there are clear seasonal variations in the occurrence of influenza, with a marked peak in cold winter months [ 2]. In contrast, seasonality is less defined in tropical regions—here, there is high background influenza activity throughout the year, on top of which epidemics occur at intermediate months between the influenza season in temperate countries of the Northern and Southern hemispheres. Influenza seasonal patterns in the Americas are summarized in Figure 1, which illustrates the transition between the Northern and Southern hemispheres. Very little is known about exactly how and why the seasonality of influenza varies with latitude. No link with environmental conditions has been convincingly shown, although influenza activity appears to coincide with the rainy season in some tropical countries [ 3].

**Figure 1:**
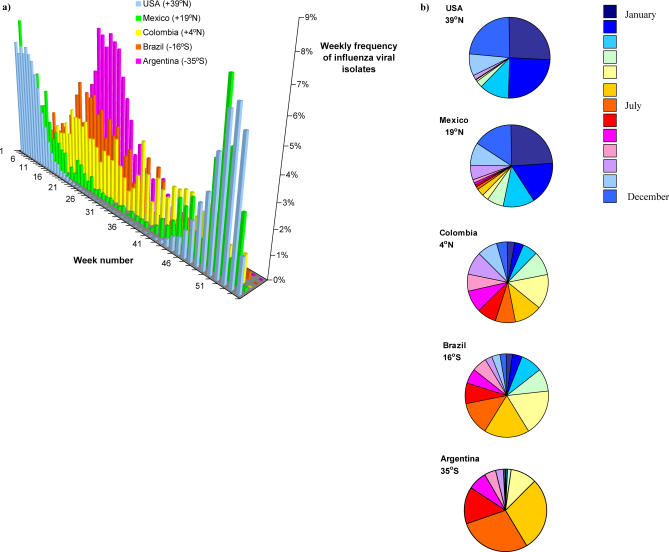
Comparison of Influenza Seasonal Patterns in Temperate and Tropical Countries in the Americas (From Lat +39°N to -35°S) The latitude of the capital city is indicated for each country in the legend (A) Weekly distribution of influenza laboratory isolates (from week 1 to week 52; weekly frequency ( *y*-axis) is calculated as the weekly number of isolates divided by the annual number of isolates). (B) Weekly numbers of isolates were aggregated over four-week periods to show a (nearly) monthly distribution of influenza circulation. Each color represents a different month (color bar on the right). Note the transition in seasonal patterns from north to south, ranging from marked seasonal winter activity centered around January in the US, to uniform circulation throughout the year in Columbia and again, strong winter epidemics center around July in Argentina. Influenza viral surveillance data compiled from WHO Flunet.

Until very recently, the impact of influenza on health was believed to be insignificant in tropical regions. But this impression may be changing, in part thanks to the recent studies by Joseph S. M. Peiris and his colleagues from Hong Kong, one of which appears in this issue of *PLoS Medicine* [ 4]. Hong Kong is a subtropical city located within the likely epicenter of pandemic influenza in Southeast Asia [ 5].

## Methods for Estimating the Burden of Influenza

Estimating the burden of influenza on hospitalizations and deaths is not straightforward in temperate countries, and it is even less so in tropical countries [ 2]. Severe clinical outcomes are often caused by secondary bacterial infections, and a primary influenza infection may be unrecognized. Additionally, laboratory confirmation of influenza infection is rarely conducted. As a result, most influenza-related hospitalizations and deaths are not attributed to influenza on discharge forms and death certificates, and statistical methods are necessary to assess the true burden of influenza on health.

Traditionally, for temperate countries, the impact of a particular influenza epidemic is estimated as the number of “excess deaths” or “excess cases” above a seasonal baseline. This approach relies on identifying periods without influenza activity to generate a model baseline of expected winter deaths (or cases) [ 6–8]. But the approach fails in tropical regions, where there is no well-defined non-influenza period ( Figure 1). An alternative method recently developed by the United States Centers for Disease Control uses virus surveillance data to guide models of burden, assuming a proportional relation between virus activity and the number of hospitalization or deaths attributable to influenza [ 9, 10]. For the US, both traditional and new methods produced very similar estimates of influenza mortality burden ( Table 1 and [ 11, 12]). In their Hong Kong study, Peiris et al. appropriately used a variation of the virus-guided modeling approach by linking weekly hospital discharges with weekly prevalence of influenza virus in respiratory specimens sampled in the community [ 4].

**Table 1:**
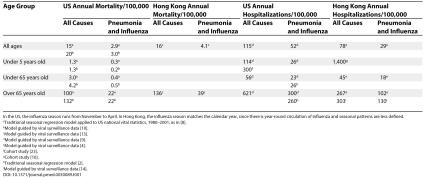
Comparison of the Average Annual Burden of Influenza on Hospitalization and Death in Recent Years in Hong Kong and the US

## Influenza Burden is Broadly Similar in Hong Kong and the US

Peiris et al. found that the annual influenza-related hospitalization rates are quite similar in Hong Kong and temperate countries ( Table 1), confirming their earlier studies [ 13, 14]. In Hong Kong, as in many other countries, the impact of influenza is seen not only on pneumonia and influenza outcomes [ 12, 15], but also on a wider range of chronic health conditions such as diabetes and cardiovascular diseases [ 12, 15]. Peiris et al. also report that influenza-related hospitalization rates vary with age as a U-shaped curve, where young infants and elderly are at highest risk of severe disease outcome, reminiscent of the age pattern of interpandemic influenza in the US and other temperate countries. However, one difference between Hong Kong and US children stands out: these authors had shown in a previous hospital cohort study that young children in Hong Kong had a hospitalization rate that was roughly 10-fold higher than in the US ( Table 1 and [ 16]). The reasons for this substantial discrepancy are elusive, but may in part be attributed to the notorious difficulty of assessing with accuracy the impact of influenza on children. There is a particularly high background rate of acute respiratory illnesses caused by other respiratory pathogens in this age group. Unfortunately, the authors did not reexamine this apparent discrepancy in the new, larger citywide study published this month [ 4]—so the question of a true regional difference in pediatric rates of influenza-related pneumonia hospitalizations remains unresolved.

## The Need for More Influenza Studies in Tropical Regions

Many research questions about influenza in tropical regions remain unanswered. For instance, Hong Kong is a wealthy subtropical city, with a higher per-capita gross domestic product than Australia or New Zealand. Thus, the present Hong Kong study cannot inform us about the impact of influenza in poor tropical countries, where the severity of infections is likely compounded by malnutrition, limited supplies of antibiotics to treat secondary bacterial infections, and limited access to hospital care.

Modeling the influenza burden in tropical countries relies on good-quality virus surveillance data. And because of large year-to-year variations in the impact of influenza, modeling also depends on the availability of several years' worth of data. Since influenza surveillance efforts were only recently initiated in tropical countries, studies of the influenza burden are generally of short duration (five years in the study by Peiris et al. [ 4]). The information generated so far during a period mostly dominated by more pathogenic influenza A (H3N2) viruses may not reflect the average influenza experience in Hong Kong [ 13]. An additional shortcoming of the virus-driven modeling approach is that it cannot study past epidemics and pandemics for which no viral surveillance data was collected. Furthermore in this approach, deaths or hospitalizations attributed to influenza are directly proportional to the intensity of virus activity, and therefore, week-to-week variations in virus isolation rates should truly reflect changes in disease prevalence and not sampling error. Laboratory surveillance needs to be strengthened before modeling of disease burden becomes feasible in most tropical countries [ 1].

Finally, more studies are needed to elucidate influenza seasonal patterns across a large range of latitudes, representing several tropical countries in both hemispheres. Of particular interest is the long-standing question of persistence: does influenza persist in temperate countries all year long (through undetectable chains of transmission in summer) or is it reintroduced from the Tropics each year at the beginning of winter? A combination of enhanced virus surveillance and sequence analysis of the viral genome of circulating strains is an important avenue for future research to address this question, and Hong Kong may be an essential sampling site in this respect.

Resolving this question is of paramount importance to vaccine composition and influenza control in tropical regions. Because of year-round influenza activity, it is not clear whether the Northern Hemisphere or Southern Hemisphere recommended composition should be used, and what the optimal timing for the vaccination campaign would be. Indeed, the influenza vaccination strategy may have to be tailored to each individual tropical region, based on local prevalence, timing, cycling of influenza strains, and how they match the strains circulating in temperate areas.

## Who Should Be Vaccinated?

Peiris and colleagues' study implies that influenza is an underappreciated public health issue in tropical regions—indeed, influenza vaccine coverage is far lower in Hong Kong than in most developed countries with a temperate climate [ 17]. The authors argue for wider use of influenza vaccines in the elderly and chronically ill; however, this strategy has not been associated with any measurable reduction in influenza-related mortality in the US [ 8], likely in part because of reduced responsiveness to the vaccine in the elderly [ 18]. In the US and Europe, moving toward universal vaccination is now being discussed as a strategy to further reduce the impact of influenza in high-risk groups by indirect means. Vaccinating schoolchildren may be especially beneficial in this regard, since they are the main spreader of influenza in schools and households [ 19–21]. If the unusually high impact of influenza in young Hong Kong children is real, then this would be an additional argument for vaccinating schoolchildren in this city, as preschoolers are likely to contract influenza from their older siblings [ 22].
